# Human papillomavirus-associated head and neck squamous cell carcinoma cells rely on glycolysis and display reduced oxidative phosphorylation

**DOI:** 10.3389/fonc.2023.1304106

**Published:** 2024-01-11

**Authors:** Nora Li, Imen Chamkha, Gaurav Verma, Sabine Swoboda, Malin Lindstedt, Lennart Greiff, Eskil Elmér, Johannes Ehinger

**Affiliations:** ^1^ Mitochondrial Medicine, Department of Clinical Sciences Lund, Lund University, Lund, Sweden; ^2^ Department of Otorhinolaryngology, Head and Neck Surgery, Skåne University Hospital, Lund, Sweden; ^3^ Department of Clinical Sciences Lund, Department of Clinical Sciences, Lund University, Lund, Sweden; ^4^ Department of Immunotechnology, Lund University, Lund, Sweden

**Keywords:** head and neck squamous cell carcinomas, human papillomavirus, mitochondria, metabolomics, glycolysis

## Abstract

**Introduction:**

Head and neck squamous cell carcinoma (HNSCC) constitutes a heterogeneous group of cancers. Human papilloma virus (HPV) is associated with a subtype of HNSCC with a better response to treatment and more favorable prognosis. Mitochondrial function and metabolism vary depending on cancer type and can be related to tumor aggressiveness. This study aims to characterize the metabolism of HPV-positive and HPV-negative HNSCC cell lines.

**Methods:**

Oxidative phosphorylation (OXPHOS) and glycolysis were assessed in intact cells, in four HNSCC cell lines using Seahorse XF Analyzer. OXPHOS was further studied in permeabilized cells using high-resolution respirometry in an Oroboros O2K. Metabolomic analysis was performed using mass spectroscopy.

**Results:**

The HPV-negative cell lines were found to display a higher OXPHOS capacity and were also able to upregulate glycolysis when needed. The HPV-positive cell line had a higher basal glycolytic rate but lower spare OXPHOS capacity. These cells were also unable to increase respiration in response to succinate, unlike the HPV-negative cells. In the metabolomic analysis, the HPV-positive cells showed a higher kynurenine/tryptophan ratio.

**Discussion:**

HPV-positive HNSCC preferred glycolysis to compensate for lower OXPHOS reserves, while the HPV-negative HNSCC displayed a more versatile metabolism, which might be related to increased tumor aggressiveness. The higher kynurenine/tryptophan ratio of HPV-positive HNSCC might be related to increased indoleamine 2,3-dioxygenase activity due to the carcinoma’s viral origin. This study highlights important metabolic differences between HPV-positive and HPV-negative cancers and suggests that future metabolic targets for cancer treatment should be individualized based on specific tumor metabolism.

## 1 Introduction

Head and neck cancer is the 7^th^ most common type of cancer worldwide, with 890 000 new cases and 450 000 deaths in 2018 (1). Out of these, head and neck squamous cell carcinoma (HNSCC) accounts for more than 90%. Current treatments for HNSCC include surgery, radiotherapy and chemotherapy. The prognosis varies greatly depending on patient age, tumor site, stage, and specific factors such as association with high-risk human papilloma virus (HPV). While HNSCC traditionally has been associated with alcohol abuse and tobacco smoking, HPV is now a rising risk factor particularly associated with tonsillar cancer. In recent years, HPV-associated HNSCC has accounted for the majority of new HNSCC cases in the Western world (2). These tumors have a better prognosis than their HPV-negative counterparts (3) due to their high responsiveness to both radiotherapy and chemotherapy (4–6). HPV-associated HNSCC is commonly caused by HPV16 (7). Integration of viral genes into the host genome, and subsequent overexpression of oncogenes is key to promoting cancer transformation of host cells (8), while also altering cellular metabolic pathways in various ways (9, 10).

Since the Warburg effect was described in 1925, mitochondrial function has been implicated in cancer biology. The Warburg effect is a propensity of cancer cells to shift towards glycolysis and away from oxidative phosphorylation (OXPHOS) for ATP production (11). However, this is often a simplification of reality. Cancer metabolism seems to vary between tumor types, between cell types within a tumor and also between different tumor microenvironments (TMEs) (12–15). Variations in cancer cell metabolism may be associated with tumor aggressiveness and patient outcome (16–18). Metabolic profiles are also linked to variations in radiosensitivity, as shown in both HNSCC (19) and other cancer types (20–22), where a shift towards glycolysis seems to induce radioresistance (23). HNSCC metabolism has been shown to predominantly involve glycolysis (24), but cancer tissues may also contain several different metabolic compartments consisting of cancer/stromal or proliferating/non-proliferating cells, which rely more or less on mitochondrial respiration (25, 26). Few studies describe differences in metabolic profiles between HPV-positive and HPV-negative HNSCC. It has been suggested that HPV-negative HNSCC is more glucose-dependent and lactate-producing, while HPV-positive HNSCC prefers mitochondrial respiration (27, 28). A better understanding of metabolism and mitochondrial function in HNSCC is warranted, especially with regard to HPV status, as HPV-positive and HPV-negative HNSCC reflect two distinctive clinical sub-groups of the disease. Metabolic differences may be an important clue in predicting disease progression and treatment response, and better mapping of key metabolic pathways could help identify possible treatment targets. In this study, we aim to describe the mitochondrial function and metabolic differences between HPV-positive and HPV-negative HNSCC cell lines.

## 2 Materials and methods

### 2.1 Cell lines

Four previously established human HNSCC cell lines were used: LU-HNSCC4 (oral cavity), LU-HNSCC5 (gingiva), LU-HNSCC6 (tongue) and LU-HNSCC26 (tonsil). The first three were established from non-HPV-associated squamous cell carcinomas (29). HNSCC26 was established from an HPV16-associated oropharyngeal carcinoma (30). HNSCC4, HNSCC5 and HNSCC6 were cultured in Dulcco’s modified Eagle’s medium (DMEM) (Sigma-Aldrich, St Louis, US) supplemented with 10% fetal bovine serum (FBS), 100 units/mL penicillin and 100 units/mL streptomycin sulphate. HNSCC26 was cultivated in RPMI 1640 with stable glutamine (Biowest, Nuaillé, France) supplemented with 10% FBS, 1 mmol/L sodium pyruvate, 1x MEM non-essential amino acids and 20 µg/mL gentamicin. As has previously been shown, HNSCC26 grows slower *in vitro* and has a three times longer doubling time compared to the other cell lines (30). This cell line was supplemented with 0.4 µg/mL hydrocortisone prior to harvest for metabolomic analysis, to time with the harvest of the other cell lines.

### 2.2 Real time metabolic assays

Cellular oxygen consumption rate (OCR) and extracellular acidification rate (ECAR) were measured with a Seahorse XF96 Extracellular Flux Analyzer (Agilent Technologies, Santa Clara, US), using XF Mito Stress Test and XF Glycolysis Stress Test. In an XF96 cell culture microplate, cells were seeded at 1.5 x 10^4^ cells/well (HNSCC4, -5, -6) and at 2.4 x 10^4^ cells/well (HNSCC26, due to more sparse growth of this cell line) in 80 µL growth medium and incubated at 37°C overnight until a monolayer was formed. The following day, XF assay medium (XF Base medium with 2 mM L-glutamine for Glycolysis Stress Test, as well as additional 10 mM glucose and 1 mM pyruvate for Mito Stress Test) was prepared and pH adjusted to 7.4. The cell plate was washed with assay medium and incubated in a 37°C non-CO_2_ incubator for one hour. 200 µL XF Calibrant was added to each well of a XF sensor cartridge which was incubated similarly. Afterwards, the injection ports were loaded with appropriate amounts of assay solutions. Fresh XF assay medium was added to the cell plate to a final volume of 180 µL per well and cell adherence was confirmed under microscope. During assay runs, basal respiration was measured followed by injection of port solutions and subsequent respiration measurements. For Mito Stress Test, 2 µg/mL oligomycin was added to inhibit ATP-synthase, followed by uncoupling of the electron transport system (ETS) by titration of carbonyl cyanide p-(trifluoromethoxy) phenylhydrazone (FCCP) using 0.5, 0.75, 1.0 and 1.25 µM in respective wells. Finally, 2 µM rotenone and 1 µg/mL antimycin-A was added to inhibit ETS, giving mitochondria-independent oxygen consumption. For Glycolysis Stress Test, 10 µM glucose was added to stimulate base glycolytic activity, followed by 2 µg/mL oligomycin to reach maximum glycolytic capacity. Finally, 50 µM 2-deoxy-D-glucose (2DG) was added to inhibit glucose hexokinase, measuring non-glycolytic acidification.

### 2.3 Data normalization

Respiration data from the Seahorse experiments were normalized to citrate synthase activity and protein content. Citrate synthase activity was analyzed using Citrate Synthase Assay Kit (Sigma-Aldrich, St. Louis, US). Cells from all cell lines were resuspended in mitochondrial respiration medium (MiR05) (sucrose 110 mM, HEPES 20 mM, taurine 20 mM, K-lactobionate 60 mM, MgCl_2_ 3 mM, KH_2_PO_4_ 10 mM, EGTA 0.5 mM, BSA 1 g/l) to a concentration of 3 x 10^6^ cells/mL, then sonicated using Ultrasonic homogenizer 4710 Series (Cole-Parmer Instrument, Vernon Hills, US). Samples were added to a 96-well plate together with 1x Assay buffer, 30 mM acetyl-coenzyme A and 10 mM 5,5’-Dithiobis-2-nitrobenzoic acid. 10 mM oxaloacetic acid was added and citrate synthase measured with BIO-RAD Microplate Reader Model 680 (Bio-Rad, Hercules, US).

Protein contents were analyzed with Bradford Protein Assay Kit II (Bio-Rad, Hercules, US). After each Seahorse experiment, the plates were washed with 1x phosphate-buffered saline and frozen at -80°C. Before assay, the cells were thawed and lysed with Radioimmunoprecipitation Assay lysis buffer. 10 µL of cell lysate was mixed with 190 µl Protein Assay Dye Reagent and transferred to assay plate. Absorbance was recorded using Microplate Reader Model 680 (Bio-Rad, Hercules, US) and compared against Bradford Assay Standards.

### 2.4 High-resolution respirometry

In-depth assessment of mitochondrial function, with substrate control for different respiratory pathways, was performed using High-resolution respirometry O2k system (Oroboros Instruments, Innsbruck, Austria). 1 x 10^6^ cells were suspended in MiR05 for each 2 mL test chamber. Basal respiration was measured, followed by subsequent injections. Digitonin was used for cell permeabilization, with optimal amount for each cell line determined as previously described (31) and found to be 12.5 µg per chamber for the HPV-negative cell lines and 15 µg per chamber for HNSCC26. After basal respiration reached steady state, digitonin, 2 mM malate and 5 mM pyruvate were added. OXPHOS was evaluated by sequential injection of 1 mM ADP, 5 mM glutamate and 10 mM succinate. After this, 10 µM cytochrome c was added with no following increase in OCR detected, demonstrating outer mitochondrial membrane integrity. 1 µg/mL oligomycin was added, followed by titration of FCCP in steps of 2 µM until no further increase in respiration. 2 µM rotenone was added to isolate respiration through complex II. Finally, 1 µg/mL antimycin-A was added. All concentrations given are final concentration in the experiment.

### 2.5 Metabolomic analysis

Cells were harvested from three different passages of each cancer cell line for metabolomic analysis. Adherent cells were washed with 5% mannitol solution twice before 800 μL methanol and 550 μL internal standard solution (H3304-1002, Human Metabolome Technologies, Tsuruoka, Japan) were added. 1 mL of the resulting solution was centrifugated at 2,300 × g at 4°C for 5 min. 350 μL of the supernatant was transferred into a centrifugal filter unit and centrifuged at 9,100 × g at 4°C until no liquid remained. Samples were evaporated in a centrifugal evaporator at 1,500 rpm, 1,000 Pa, and kept at -80°C until shipping.

Metabolomic analysis was performed by Human Metabolome Technologies Inc. (Tsuruoka, Japan). Cells were reconstituted in Milli-Q water and analyzed using the cation and anion modes of the capillary electrophoresis Fourier transform mass spectrometry (CE-FTMS) (Agilent Technologies, Santa Clara, US), performed as previously described (32, 33). Peaks detected in the CE-FTMS analysis were extracted using automatic integration software (MasterHands ver. 2.19.0.2 developed at Keio University) to obtain peak information, which includes m/z, migration time (MT) and peak area. The peak area was converted to a relative peak area. Putative metabolites were assigned from HMT’s standard library based on m/z and MT. The tolerance was ±0.5 min in MT and ±5 ppm in m/z. All metabolite concentrations were calculated by normalizing the peak area of each metabolite concerning the area of the internal standard and by using standard curves, which were obtained by single-point (10 μM) calibrations.

### 2.6 Statistics

Statistical evaluation was performed using GraphPad PRISM version 9.1.1 (GraphPad Software, Boston, US). Data were presented as means ± standard deviations (SD). Comparisons of experimental data were made using one-way ANOVA followed by Tukey’s multiple comparisons test. A p-value <0.05 was considered statistically significant. Metabolomic data were analyzed using Welch’s t-test and plotted using volcano plots for HPV-positive against HPV-negative SCC cell lines, and for HNSCC26 against each of the HPV-negative cell lines. A 1-fold difference between data sets and a p-value <0.01 was used as a cut-off.

## 3 Results

**Figure 1:**
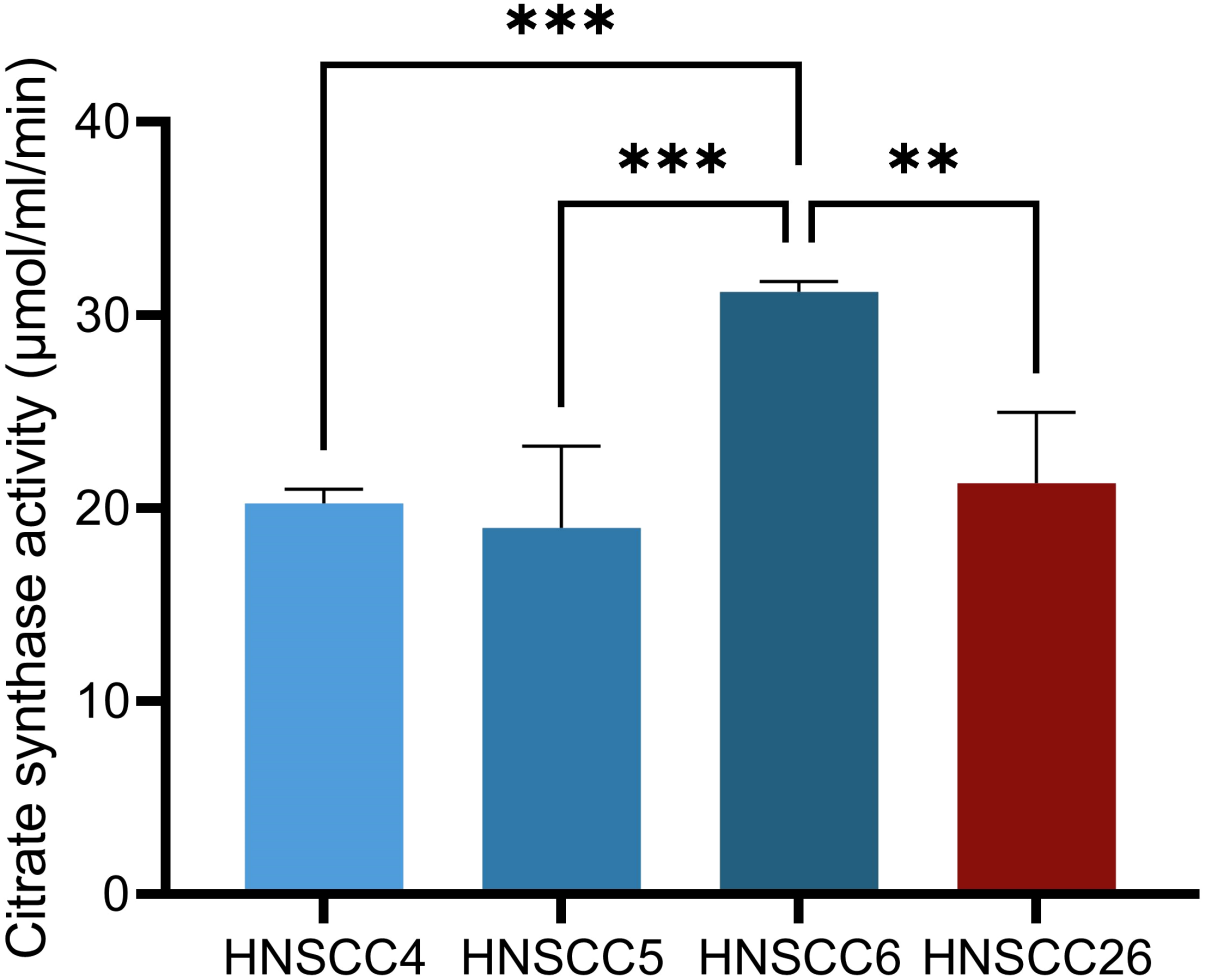
Citrate synthase activity in HNSCC cell lines. HNSCC6 exhibited higher activity than the other cell lines. Data analyzed with one-way ANOVA followed by Tukey’s multiple comparisons test, presented as means with error bars representing standard deviations. *n* = 4, **p < 0.010, ***p < 0.001. (HNSCC, Head and Neck Squamous Cell Carcinoma).

Four cell lines derived from squamous cell carcinomas were used in this study (**Table 1**), three HPV-negative and one HPV-positive. Analysis of citrate synthase activity showed higher citrate synthase activity for HNSCC6, while the activities for the remaining cell lines were similar to each other (**Figure 1**), suggesting that HNSCC6 had a higher mitochondrial content. Microscopically, HNSCC26 differed by having a flatter, spread-out appearance and a sparser growth pattern. Similar to previous observations on this cell lines, we observed a slower growth rate for HNSCC26 (30).

**Table 1:** Head and neck squamous cell carcinoma (HNSCC) cell lines and characteristics of the primary tumors [data from previously published studies (29, 30)].

	HNSCC4	HNSCC5	HNSCC6	HNSCC26
HPV status	–	–	–	+
Origin localization	Oral cavity	Gingiva	Tongue	Tonsil
Disease free months	37	4	6	No relapse
Overall survival months	42*	5	6	N/A

*Patient death due to intercurrent disease.

### 3.1 Mitochondrial and glycolytic function

**Figure 2:**
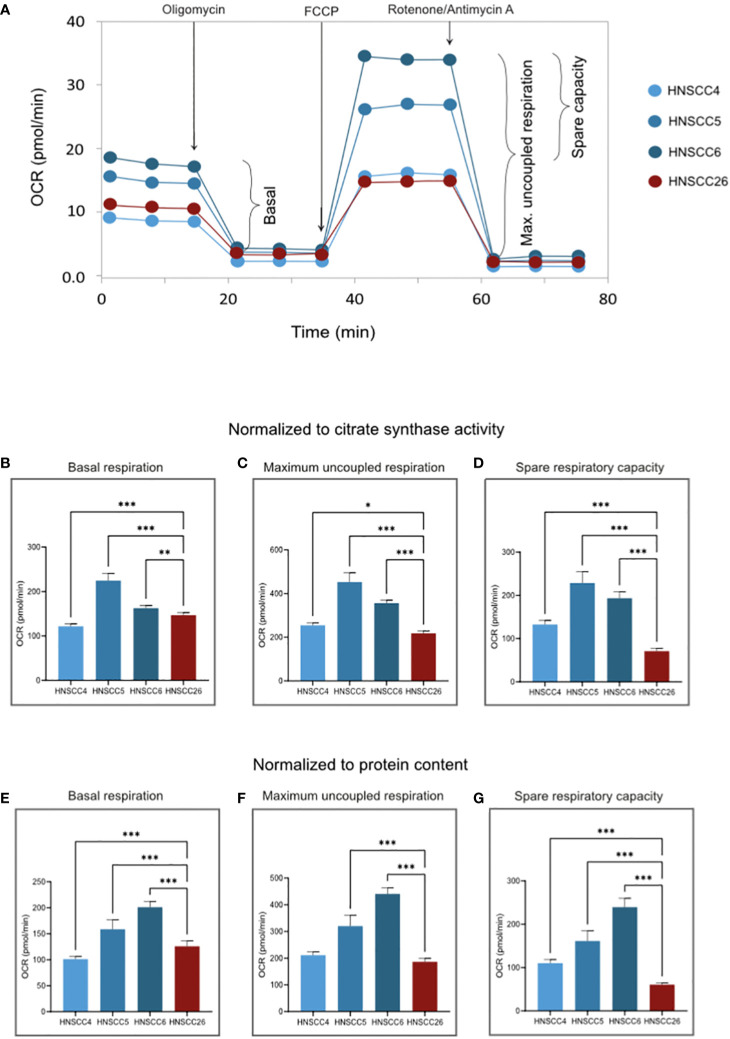
OXPHOS of HNSCC cell lines. **(A)** Representative experimental traces from a Seahorse XF Analyzer for each cell line, with additions of inhibitors/uncouplers and measured respiratory states indicated. **(B–D)** OCR normalized to citrate synthase activity. **(E–G)** OCR normalized to the protein content of each test plate (**Supplementary Figure 1**). Basal respiration rate is similar between cell lines. HNSCC26 exhibited lower spare respiratory capacity than the HPV-negative cell lines. Data analyzed with one-way ANOVA followed by Tukey’s multiple comparisons test, presented as means with error bars representing standard deviations. *n* = 8, *p < 0.05, **p < 0.010, ***p < 0.001. (HPV, Human Papilloma Virus; HNSCC, Head and Neck Squamous Cell Carcinoma; OCR, Oxygen Consumption Rate; OXPHOS, Oxidative Phosphorylation).

**Figure 3:**
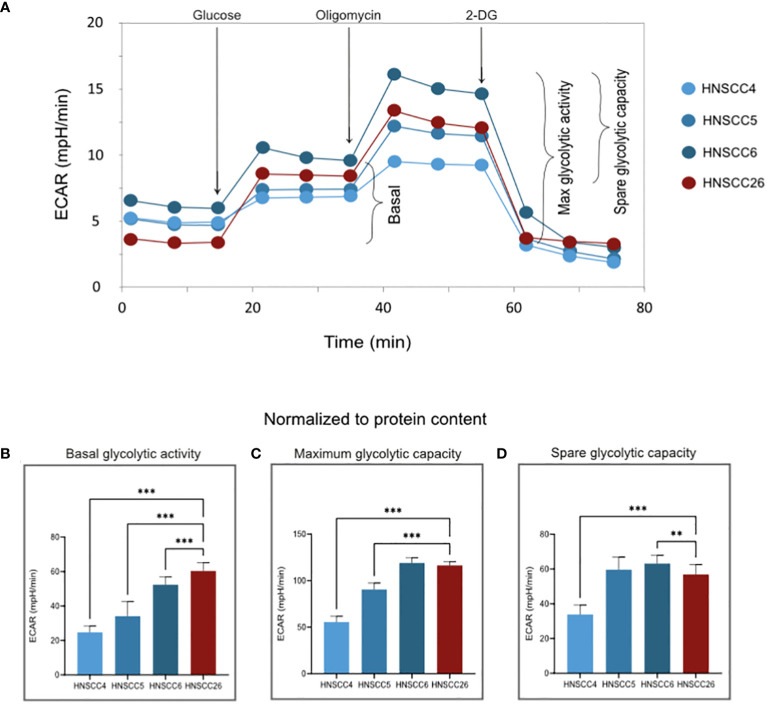
Glycolytic activity of HNSCC cell lines **(A)** Representative experimental traces from a Seahorse XF Analyzer for each cell line, with additions of inhibitors/glucose and measured respiratory states indicated. **(B–D)** ECAR normalized to the protein content of each test plate (**Supplementary Figure 1**). Basal glycolytic activity was higher in HNSCC26 but all cell lines were able to increase glycolysis in response to substrate addition. Data analyzed with one-way ANOVA followed by Tukey’s multiple comparisons test, presented as means with error bars representing standard deviations. *n* = 8, **p < 0.010, ***p < 0.001.

OXPHOS and glycolysis were analyzed using two different Seahorse protocols, where OXPHOS was measured as oxygen consumption rate (OCR) and glycolysis as extracellular acidification rate (ECAR) (**Figures 2A**, **3A**). To characterize metabolism in relation to both mitochondrial content and cell number, OXPHOS data was normalized using citrate synthase activity and protein content. Glycolysis data was normalized only using protein content, since mitochondrial content has limited relevance as a normalization factor for glycolytic activity. Bradford protein assay showed very similar protein content levels in all four cell lines (**Supplementary Figure 1**).

For OXPHOS, basal ATP-linked respiration and the maximum capacity of the ETS were measured (the resulting difference between these two values often referred to as the spare respiratory capacity). HNSCC5 and HNSCC6 exhibited significantly higher activity (p < 0.001), with higher basal respiration as well as higher maximum respiratory capacity (**Figures 2B, C**). HNSCC4 and HNSCC26 were more alike in basal respiration, but HNSCC26 was unable to increase to similar maximum respiration. In particular, HNSCC26 showed a significantly lower spare respiratory capacity in response to maximum uncoupling (p < 0.001) (**Figures 2D, G**). Within the HPV-negative group, HNSCC6 showed the highest OCR normalized to protein content (**Figures 2E–G**). In contrast, when normalized to citrate synthase activity, the OCR of HNSCC5 was higher both in basal and uncoupled respiration (**Figures 2B–D**). HNSCC26 on the other hand consistently showed lower maximum and spare respiratory capacity.

With regard to glycolysis, HNSCC26 exhibited the highest basal activity with higher ECAR than all other cell lines (p < 0.001) (**Figure 3B**). HNSCC26 also showed a relatively high spare glycolytic capacity (**Figure 3D**), contrasting with its limited oxidative respiratory capacity. All cell lines were able to increase their glycolytic rate in response to the added substrate (**Figures 3C, D**).

### 3.2 High-resolution respirometry

**Figure 4:**
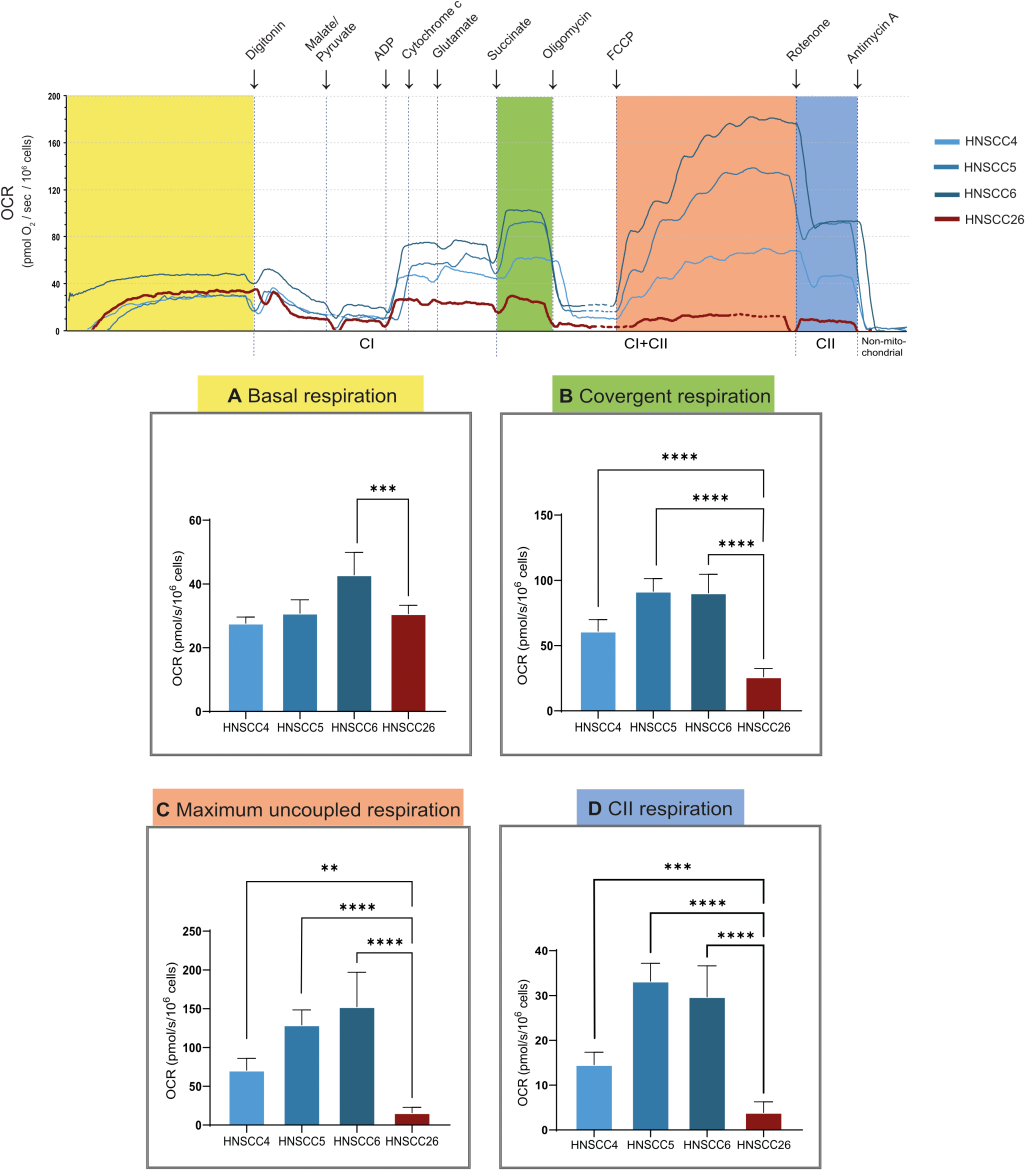
High resolution respirometry of HNSCC cell lines, using Oroboros O2k. Representative trace is shown, oxygen consumption rate (OCR) is measured at base level and after the addition of substrates, inhibitors and uncoupler. **(A)** Basal respiration. **(B)** Respiration after cell permeabilization in presence of CI and CII-substrates and ADP. HNSCC26 was unable to increase respiration in response to succinate, in contrast to the HPV-negative cell lines. **(C)** Respiration after maximum uncoupling of ETS. All cell lines responded to uncoupling except for HNSCC26. **(D)** CII-linked respiration. Data analyzed with one-way ANOVA followed by Tukey’s multiple comparisons test, presented as means with error bars representing standard deviations. *n* = 7, **p < 0.010, ***p < 0.001, ****p < 0.0001.

High-resolution respirometry was performed using permeabilized cells for closely controlled substrate conditions to enable a detailed assessment of mitochondrial function. Again, HNSCC6 exhibited higher basal respiration compared to the other cell lines (**Figure 4A**). After cell permeabilization, the addition of NADH-linked substrates led to a sharp increase in complex I (CI) linked respiration. This could be seen in all cell lines; however, for HNSCC26 the CI-linked respiration was lower despite similar initial basal respiration. After the addition of succinate, all HPV-negative cell lines showed an increase in oxygen consumption as a sign of induced complex II (CII) linked respiration. This was especially prominent for HNSCC5 and HNSCC6. HNSCC26 on the other hand was unable to increase respiration in response to succinate compared to the other cell lines (p < 0.0001) (**Figure 4B**). In response to maximum uncoupling, all HPV-negative cell lines displayed increased convergent respiration (CI and CII-linked). This contrasted to HNSCC26 which had virtually no response to uncoupling and consequently had a significantly lower maximum uncoupled respiration (p < 0.0001) (**Figure 4C**). Isolation of CII activity also showed much lower activity in HNSCC26 (p < 0.0001) (**Figure 4D**).

### 3.3 Metabolomic analysis

**Figure 5:**
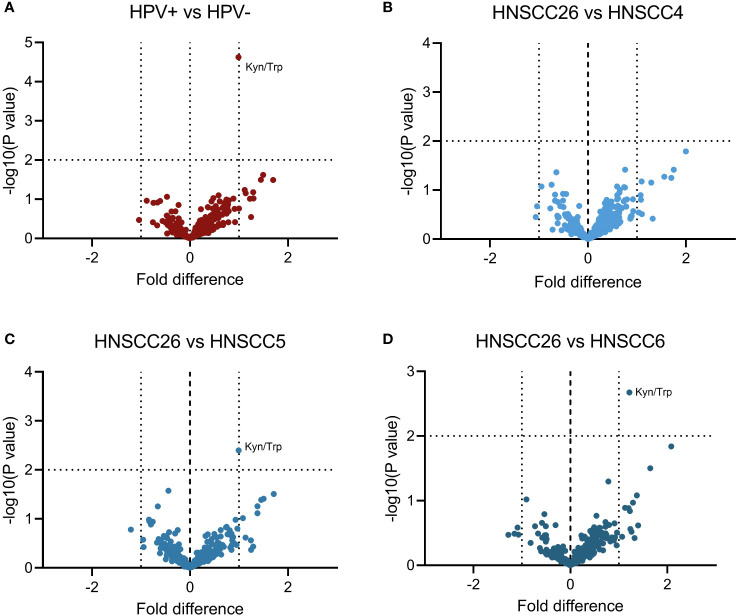
Comparison of metabolites between HNSCC cell lines. A total of 517 metabolites were detected in metabolomic analysis and included. **(A)** HPV-positive cell line (HNSCC26) compared to all HPV-negative cell lines. **(B–D)** HNSCC26 compared to HNSCC4, HNSCC5, and HNSCC6 respectively. Kynurenine/tryptophan ratio (Kyn/Trp) was increased in HNSCC26. Data analyzed using Welch’s t-test, a 1-fold difference and p < 0.010 was considered statistically significant (*n* = 3).

**Figure 6:**
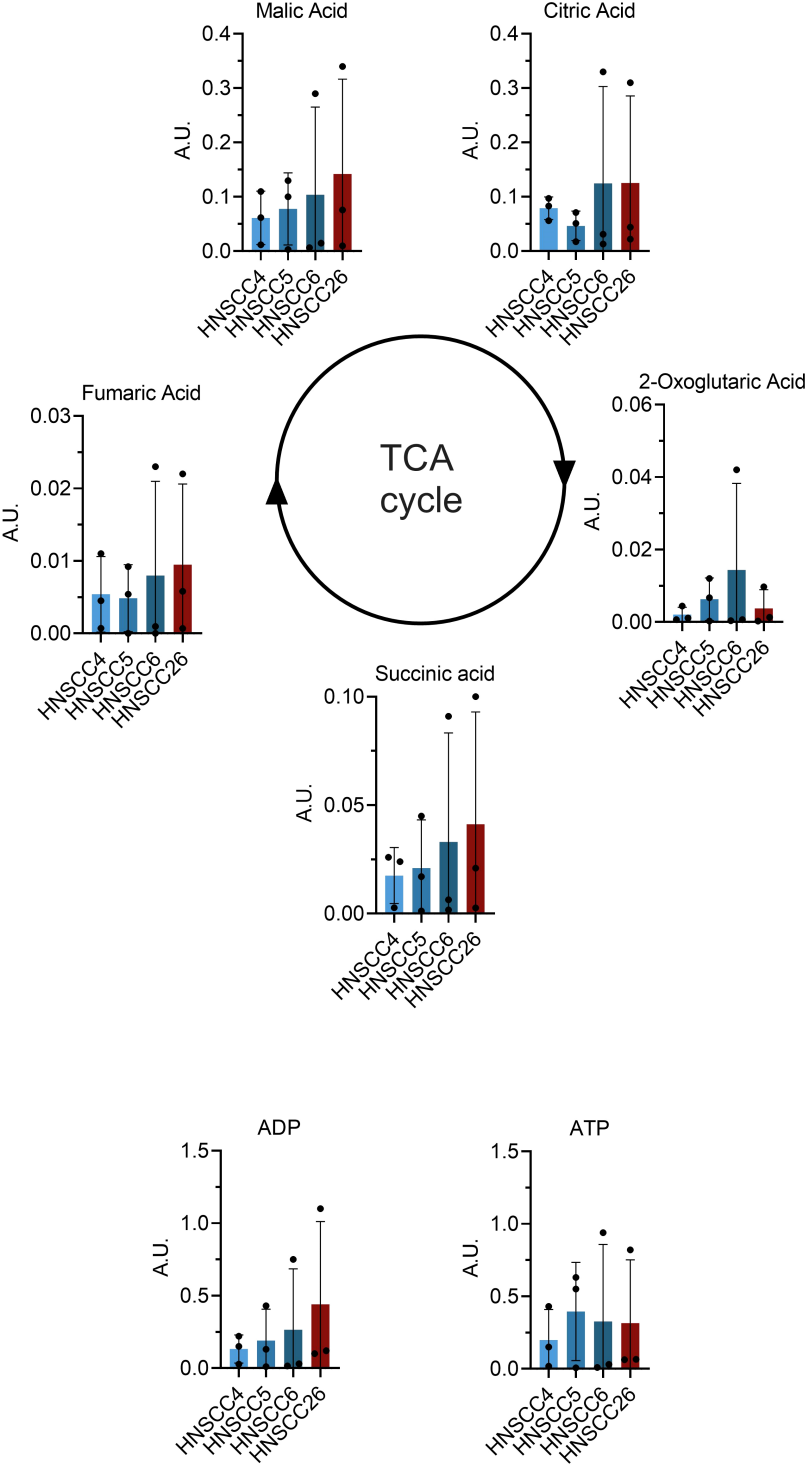
Comparison of tricyclic acid (TCA) cycle metabolites between HNSCC cell lines. Data from metabolome analysis presented as means with error bars representing standard deviations (*n* = 3). No significant differences were found between the metabolites of the cell lines.

Metabolomic analysis was carried out on all four cell lines, and in total 517 metabolites were detected. Inter-group comparisons of metabolites showed one consistent difference. Kynurenine/tryptophan ratio (Kyn/Trp) was higher in HNSCC26 compared to the HPV-negative cell lines as a whole (p < 0.0001) (**Figure 5A**), as well as compared to HNSCC5 and HNSCC6 separately (**Figures 5B–D**). While the respirometry data demonstrated metabolic differences between the HPV-positive and HPV-negative cell lines, in the metabolomic analysis of the cell lines we were unable to find any significant differences in metabolites involved in glycolysis or the tricyclic acid cycle (TCA) (**Figure 6**).

## 4 Discussion

Studies on energy metabolism in cancer show that cancer cells are widely diversified with many different metabolic needs, which was also demonstrated in this study involving cell lines derived from patients with head and neck cancer. A key finding was that the HPV-positive cell line displayed an overall lower metabolic capacity and low spare respiratory capacity, which was partially compensated for by glycolysis. This result contrasts with several previous studies that indicate that HPV-positive cancers rely on mitochondrial respiration (27) through the induction of OXPHOS by HPV-specific oncoproteins (34), while HPV-negative cancers generally display a Warburg phenotype favoring glycolysis (24, 28). Furthermore, in our study, the HPV-positive cells did not upregulate respiration in response to succinate. Our results further highlight that the Warburg effect is not universally applicable to all cancers and nuances the previously proposed metabolic phenotypic difference between HPV-positive and HPV-negative cancer cells. This has a potentially high clinical relevance, as metabolic differences have been shown to affect radiosensitivity (35) as well as disease progression (36).

All four cell lines in this study had functioning mitochondrial metabolism, but differed in their metabolic profiles. HNSCC6 and HNSCC5 had a high respiratory capacity, while HNSCC4 and HNSCC26 were less metabolically active. Citrate synthase activity was the highest in HNSCC6, which points to more active mitochondria, possibly accounting for its high respiratory capacity. The HPV-negative cell lines in this study did not prefer glycolysis over OXPHOS as expected from previous reports. However, they were able to upregulate glycolysis in response to increased substrates, showing a versatile metabolic profile and a high capacity for both types of metabolism. HNSCC26 exhibited the lowest spare respiratory capacity but highest basal glycolytic rate out of all cell lines, with a high spare glycolytic capacity. Taken together, HNSCC26 prefers glycolysis over OXPHOS for energy production.

Interestingly, in high-resolution respirometry, HNSCC26 displayed a very distinctive behavior. Instead of responding to succinate addition after cell permeabilization, HNSCC26 was unable to raise respiration levels, even after maximum uncoupling. This was in contrast to the HPV-negative cell lines, which all responded well to succinate and ETS uncoupling. Optimal digitonin volume was determined for each cell line and outer mitochondrial membrane integrity was tested during high-resolution respirometry, demonstrating that these results were not due to limited permeabilization or mitochondria damage by the cell permeabilization process.

The inability to respond to succinate may point to an impaired function of succinate dehydrogenase (SDH) in HNSCC26. Succinate is linked to cancer as an oncometabolite, and mutations in its metabolizing enzyme are linked to several types of cancers (37, 38) by promoting glycolysis, angiogenesis and tumor growth (39). Tumor cells can also release succinate extracellularly, where it signals through the succinate receptor (coded by *SUCNR1*) to promote tumor-associated macrophage polarization as well as cancer cell invasion and metastasis (40). The connection between succinate and HNSCCs is so far not well established. However, one study found increases in serum succinate in HNSCC patients as well as increases in expression of *SUCNR1* and SDH in cancer cells and surrounding stroma (41). If SDH was indeed defective in HNSCC26, metabolomic analysis would be expected to reveal an increased intracellular succinate concentration, which was not the case. A possible explanation to this is succinate secretion by the tumor cells, maintaining normal intracellular succinate levels, but this needs to be confirmed in further studies.

Comparing patient data for the cell lines in this study, the tumors used to establish HNSCC5 and HNSCC6 were more clinically aggressive than the ones giving rise to HNSCC4 and HNSCC26 (29, 30). Patients for both HNSCC5 and HNSCC6 had much shorter tumor-free periods and overall survival (**Table 1**). In contrast, the patients for HNSCC4 and HNSCC26 had a tumor-free period of 37 months or no recurrence at a 4-year follow-up respectively. HNSCC26 was also previously shown to be significantly more sensitive to radiotherapy and chemotherapy (30). Interposing this with the metabolic data, we suggest the possibility that the more aggressive tumors are characterized by overall higher mitochondrial respiration, but also by a more versatile metabolism where the cells can switch to glycolysis in response to substrate changes. In a recent study, radioresistant HPV-negative HNSCC cells were found to have a greater capacity for OXPHOS (42), in contrast with precedent findings (27, 28). Similarly, we found our HPV-negative cell lines to diverge from the typical Warburg profile of aggressive tumors. Instead, it was the HPV-positive cells that used glycolysis in favor of a seemingly reduced mitochondrial respiration. Speculatively, lower mitochondrial function and low metabolic reserves might be an additional explanation for the reduced aggressiveness of HNSCC26.

HPV is described to regulate metabolism in favor of OXPHOS over glycolysis through the activity of oncoproteins E6 and E7 (9) which promotes a glycolytic phenotype (43). A 2021 study shows that in HPV-positive HNSCCs with preserved wild-type *TP53*, metabolic profiles vary and the cells exhibit a high capacity for both OXPHOS and glycolysis (44). HNSCC26 is reported to have wild-type *TP53*, but the homozygous arginine type that is more susceptible to degradation (30). One explanation for our respiratory findings may be that the p53 function is reduced in this particular cell line, but this claim needs further studies to be supported.

To comprehensively map the metabolic pathways of the four cell lines, metabolomic analyses were performed. Because of the metabolic differences between the cell lines, we expected to find differences in key metabolites of glycolysis and the TCA cycle. However, no significant differences were observed. Nonetheless, we found an increased kynurenine/tryptophan (Kyn/Trp) ratio in HNSCC26 compared to HNSCC5 and HNSCC6. An increased Kyn/Trp ratio is linked to the activation of indoleamine 2,3-dioxygenase (IDO), leading to the degradation of tryptophan to kynurenine and subsequent immunosuppression by silencing of T- and NK-cells (45). Increased IDO activity and Kyn/Trp ratio have been shown in several types of cancers, often correlating to more advanced disease and poor prognosis (46–48). With regard to HPV, increased IDO activity and Kyn/Trp ratio have been detected in HPV-positive cervical cancers, also here correlating to more advanced disease (46, 49, 50). It is theorized that the HPV-induced oncoproteins E6/E7 may be involved in the activation of IDO (49). However, there are no current studies indicating the role of IDO and the kynurenine pathway in HPV-induced HNSCC. Our results suggest that HPV-positive HNSCC may have an altered tryptophan metabolism, the implications of which warrant further investigation.

We acknowledge the inherent drawback of research on cultured cancer cells in monolayers, as they do not reflect the complexity of a malignant tumor. Notably, these cells lack a representative TME, which may alter cellular behavior and metabolic pathways in a significant way. For example, studies have shown that stromal cells are driven to switch to aerobic glycolysis which creates energy substrates for cancer cells (26, 51). When characterizing metabolism of cultured cell lines, this synergy is lost, and the results may not correspond to metabolism *in vivo*. Furthermore, we acknowledge the limited number of cell lines examined in this study, and the large variations and small sample size of the metabolomic data. Since the cell lines used in this study arose from different histological origins, this could have contributed to some of the metabolic differences. Despite this, we found distinct differences in the metabolic profile of the HPV-positive HNSCC26 that are different from the HPV-negative group.

In conclusion, we observed clear differences between the metabolic profiles of the HNSCC cell lines examined in this study. Notably, HPV-positive cells displayed an overall low metabolic capacity, with low OXPHOS partially compensated for by glycolysis. The HPV-negative cell lines had more active respiration and were capable of switching to glycolysis if needed. HPV-positive cells exhibited signs of higher IDO activity, which might be due to their viral association. Further studies are warranted focusing on the respiratory capacity of HNSCC cells in relation to other features of the TME, including its immune cell presence/activity, and to clinical characteristics of HNSCC.

## Data availability statement

The raw data supporting the conclusions of this article will be made available by the authors, without undue reservation.

## Ethics statement

Ethical approval was not required for the studies on humans in accordance with the local legislation and institutional requirements because only commercially available established cell lines were used.

## Author contributions

NL: Data curation, Formal analysis, Methodology, Writing – original draft, Writing – review & editing. IC: Writing – review & editing. GV: Data curation, Writing – review & editing. SS: Resources, Writing – review & editing. ML: Formal analysis, Resources, Writing – review & editing. LG: Formal analysis, Resources, Supervision, Writing – review & editing. EE: Conceptualization, Methodology, Resources, Supervision, Writing – review & editing. JE: Conceptualization, Formal analysis, Funding acquisition, Methodology, Resources, Supervision, Writing – review & editing.
