# Supplementary material for: Human papillomavirus-associated head and neck squamous cell carcinoma cells rely on glycolysis and display reduced oxidative phosphorylation
Source: Front Oncol. 2024 Jan 11;13:1304106. doi: 10.3389/fonc.2023.1304106 (PMC10808639; doi:10.3389/fonc.2023.1304106)
Supplement: Supplementary file 1 [file DataSheet_1.pdf]

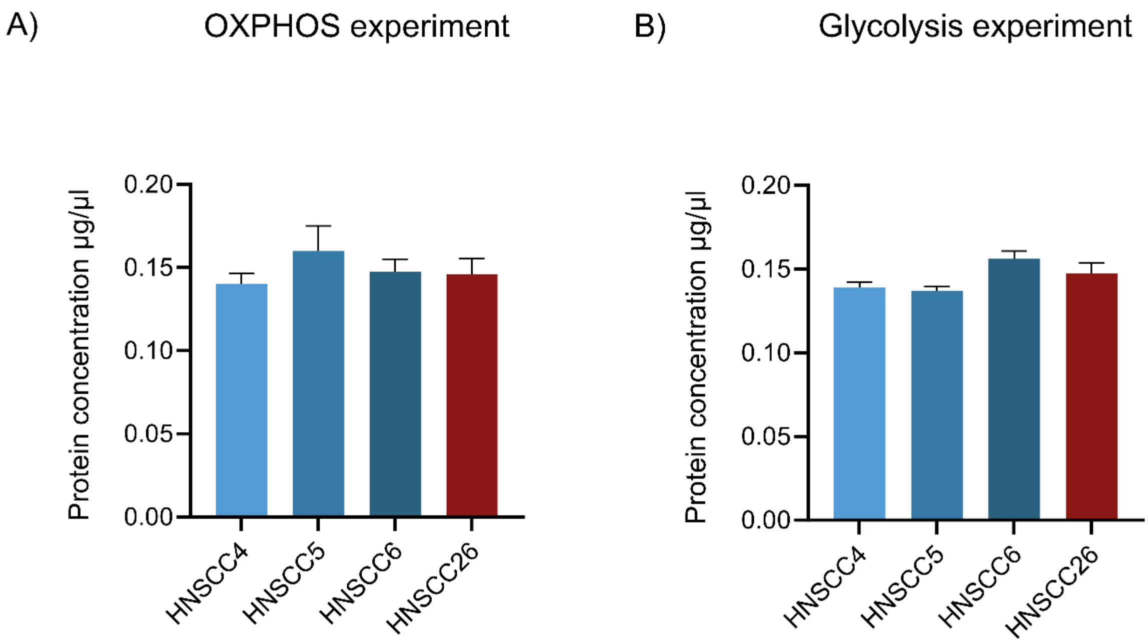

Suppl fig. 1 Protein content of test plates from Seahorse XF Analyzer experiments of HNSCC cell lines showing very similar levels between the four cell lines. (HNSCC, Head and Neck Squamous Cell Carcinoma)
